# A Cross-Sectional Seroepidemiological Study on Infectious Bursal Disease in Backyard Chickens in the Mymensingh District of Bangladesh

**DOI:** 10.1155/2022/9076755

**Published:** 2022-07-20

**Authors:** Sharmin Akhter Mili, Md. Saiful Islam, Abdullah Al Momen Sabuj, Zobayda Farzana Haque, Amrita Pondit, Md. Golzar Hossain, Jayedul Hassan, Sukumar Saha

**Affiliations:** Department of Microbiology and Hygiene, Faculty of Veterinary Science, Bangladesh Agricultural University, Mymensingh 2202, Bangladesh

## Abstract

Infectious bursal disease (IBD) is a highly contagious disease that causes significant economic loss in chickens. A cross-sectional study was conducted in the Mymensingh district of Bangladesh to determine the seroprevalence of IBD virus (IBDV) antibodies in backyard chickens and their association with different epidemiological risk factors. A total of 460 serum samples were randomly collected from backyard chickens that had not been previously vaccinated against IBDV. The collected sera were examined using an enzyme-linked immunosorbent assay (ELISA). Data on epidemiological risk factors were collected through face-to-face interviews with owners and subjected to both uni- and multivariable risk analyses to determine their association with IBDV infection. Using ELISA, the overall seroprevalence of IBDV antibodies in backyard chickens was 83.4% (95% confidence interval: 79.8%–86.6%), among which, a significantly higher seroprevalence was recorded in females (83.4%, 345/350), 4–6 weeks age group (95.3%, 244/256), and unhealthy (95.0%, 57/60) backyard chickens than those of males, other age groups, and healthy chickens, respectively. Furthermore, chickens reared in free-ranging housing systems (93.3%, 280/300) and poor-conditioned houses (98.0%, 147/150) showed a significantly higher seropositivity of IBDV antibodies than those reared in separated housing systems and other hygienic-conditioned houses, respectively. Moreover, compared with their counterparts, a higher but nonsignificant seroprevalence of IBDV antibodies was observed in backyard chickens that were selected from Fulbaria Upazila (88.8%; 80/90) and which were brought from the marketplace (85.7%, 60/70). A higher seropositivity of IBDV antibodies was shown to be statistically associated with various critical epidemiological risk factors, indicating that field strains of IBDV were exposed in backyard chickens and could be readily transferred horizontally. Proper prevention and control methods, villagers' awareness of IBD, and the rapid and widespread use of seroepidemiological investigations could help to reduce the spread of IBDV infection in backyard chickens.

## 1. Introduction

In Bangladesh, the poultry sector has developed into a thriving agricultural enterprise that significantly contributes to the country's total gross domestic product [1, 2]. Some indigenous chicken varieties are commonly called backyard chickens that are well adapted to local environments as they are excellent foragers, better able to avoid predator attacks, and demonstrate better immunity to common diseases [3]. In addition, backyard chickens in Bangladesh maintain the backbone of rural economics and the health status of rural individuals by providing additional economic support and quality protein supplementation [4, 5]. Approximately, 80% of rural individuals raise backyard chickens in free-range scavenging systems or in separate houses [6, 7]. However, the emergence of a variety of infectious diseases owing to inadequate production system practices makes it difficult to further boost their predicted output [8]. Backyard chickens are extremely sensitive to infectious viral diseases owing to their wide access to and direct contact with wild birds [9]. Furthermore, the establishment of large-scale poultry (exotic) farms in rural areas can increase the risk of contagious viral diseases spreading to conventionally handled chickens [10]. Infectious bursal disease (IBD), the second most common viral disease in poultry, causes enormous economic losses, and thus has a negative influence on the poultry sector [11, 12].

IBD, often known as Gumboro's disease, is a highly contagious immunosuppressive viral disease that affects young chickens aged 3–6 weeks and even up to 8 weeks [13]. The disease is characterized by severe lesions in the bursa of Fabricius, bursal atrophy, immunosuppression, and even mortality [14, 15]. In addition, a complication of IBD with other poultry diseases (e. g., mycoplasmosis, *E. coli* infection, and *Ornithobacterium* infection) may hasten the effects of IBD, resulting in devastating repercussions for chickens [16]. Chickens, among other domestic poultry, are natural hosts for IBD. The fecal-oral route is the most common mode of transmission of the causative agent of IBD, followed by the aerosol method [17]. IBD can affect both commercial and backyard chickens in a commensurate way [18]. Furthermore, various risk factors, including location, age, sex, health status, source, and housing system, are linked to the development of IBD in chickens [19, 20].

IBD is caused by the IBD virus (IBDV), which was first described in 1931 [21]. IBDV is a bisegmented double-stranded RNA virus belonging to the *Birnaviridae* family and *Avibirnavirus* genus with a 60 nm icosahedral nonenveloped capsid [22]. Only serotype-1, out of the two reported unique serotypes of IBDV, is highly harmful to chickens and is further divided into several variants based on antigenicity and virulence [22]. Conversely, serotype-2 can affect chickens, although it does not cause clinical diseases because it is naturally avirulent [23]. Furthermore, owing to the highly resistant nature of IBDV, if a chicken farm is contaminated once, reinfection may possibly occur [20]. These chickens can also act as carriers, spreading the disease to other commercial and exotic birds [24].

A variety of serological assays are available to identify IBDV antibodies, including enzyme-linked immunosorbent assay (ELISA), immunofluorescence test, agar gel immunodiffusion test, and viral neutralization test [25]. ELISA has been recognized as the most sensitive test for detecting IBDV antibodies in chickens [26].

Although IBD is a highly contagious disease in chickens and has a significant negative impact on the country's poultry sector, there is still a scarcity of studies on the seroepidemiological occurrence of IBD in backyard chickens in Bangladesh. This requires a thorough investigation of its epidemiological characteristics.

Considering the current important situation, we conducted the present study with the following objectives:

- To detect IBDV antibodies in backyard chickens in the Mymensingh district of Bangladesh.
- To evaluate the seroepidemiological risk factors for IBD in backyard chickens in the study area.

## 2. Materials and Methods

### 2.1. Ethical Statement

The present study was conducted with the approval of the Institutional Ethical Committee (Animal Welfare and Experimentation Ethics Committee) of Bangladesh Agricultural University, Mymensingh-2202 (AWEEC/BAU/2019[57]). During sample collection, expert veterinarians collected blood samples from the birds while keeping ethical norms and animal welfare concerns in mind. Moreover, poultry owners provided their approval verbally before sample collection.

### 2.2. Study Area Selection

**Figure 1:**
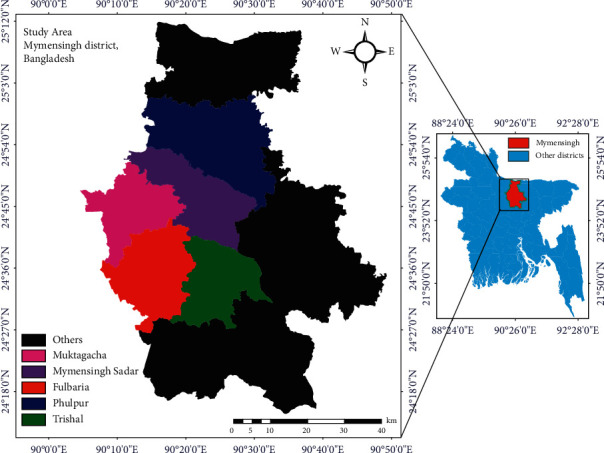
Sampling area map of the selected Upazilas of Mymensingh district, Bangladesh. Images were extracted from DIVA-GIS (https://www.diva-gis.org/) and provided by the Geographical Information System (GIS). Finally, the map was created using ArcMap software 10.7 (ArcGIS Enterprise, ESRI, Redlands, California, USA).

From June 2018 to July 2019, the present cross-sectional study was conducted in five Upazilas in the Mymensingh district of Bangladesh: Mymensingh Sadar (24.7851°N, 90.3560°E), Fulbaria (24.6251°N, 90.2665°E), Muktagacha (24.7660°N, 90.2561°E), Phulpur (24.9545°N, 90.3629°E), and Trishal (24.5817°N, 90.3949°E) Upazilas (Figure 1). Mymensingh district was selected owing to its geographical location, the presence of a large backyard chicken population, and husbandry practices adopted by the owners.

### 2.3. Sample Size Calculation

The sample size was calculated using a standard formula derived by Thrusfield [27]:

(1) $$\begin{array}{c} n=\frac{Z^{2}pq}{d^{2}}. \end{array}$$

Here, *n* = desired sample size, *Z* = standard normal deviation corresponding to the desired confidence level (1.96 at 95% confidence level), *p* = prevalence (here, we predict 50% or 0.5), *q* = 1 − *p*, and *d* = 5% desired precision. Using this formula, the estimated number of samples required was approximately 384 for backyard chickens in the Mymensingh district of Bangladesh. We expected a 15% nonresponse rate to adjust the sample size for nonresponses. Finally, we used the following formula [28] to acquire the final sample size:

(2) $$\begin{array}{c} \text{Final sample size}=\frac{\text{Estimated sample size}}{1-\text{Non}-\text{response rate estimated}}. \end{array}$$

Therefore, the final adjusted sample size was 384/(1–15%) = 384/0.85 ≈ 452. Therefore, a total of 460 backyard chickens were selected for the sample collection in the present study.

### 2.4. Epidemiological Data Collection

A structured questionnaire was used to collect epidemiological data from backyard chicken owners in 60 households (20 households from Mymensingh Sadar Upazilas and 10 each from other Upazilas). These households were chosen for the current study based on their previous experience in raising backyard chickens, the number of chickens they currently keep, and their willingness to participate in the study. Data were obtained through face-to-face interviews with owners. The questionnaire was written in English; however, it was translated into their dialect during the interview so that the farmers could comprehend the content. Three expert veterinarians, two expert microbiologists, and one trained enumerator participated in the face-to-face interview data gathering phase. The questionnaire included the owner's address and name, farming house location, morbidity, mortality, vaccination history, and clinical features of birds; however, eight variables, such as location, sex, age, breed, health status, source of backyard chickens, their housing systems, and the hygienic conditions of houses, were mainly focused during data collection.

### 2.5. Sample Collection

During sample collection, only unvaccinated backyard chickens were selected. Blood and sera were collected from backyard chickens using a method previously described by Islam et al. [29]. In brief, using a 3 mL sterile plastic syringe (PolyLab, India), 2–3 mL of blood was aseptically extracted from the wing veins of the selected birds. To induce clotting, blood-containing syringes were maintained at room temperature for 1-2 h in a standing position. Sera were collected in Eppendorf tubes (0.5 mL) (PolyLab, India) with individual tag numbers and transported in a cool chain to the Virology Laboratory, Department of Microbiology and Hygiene, Bangladesh Agricultural University, Mymensingh, Bangladesh. Immediately after transportation, the sera were centrifuged at 3,000 rpm for 5 min to remove red blood cells and insoluble debris, followed by meticulous separation of clear sera, transfer to new sterile Eppendorf tubes (0.5 mL), and storage at −20°C for further analysis.

### 2.6. Serological Analysis

Indirect ELISA was used to detect IBDV-specific antibodies in collected and processed sera. The IBDV antibody test kit (ID Screen® IBD Indirect, ID Vet, Grabels, France), including IBDV antigen-coated plates, was used for indirect ELISA. The sensitivity and specificity of the test kits used in this study were both 100%. The indirect ELISA was performed according to a previously described procedure [30].

The sample-to-positive (S/P) ratio and antibody titer were calculated as follows:

(1) For the S/P ratio,
  (3) $$\begin{array}{c} \frac{S}{P}=\frac{\text{OD of sample }-\text{OD of negative control}}{\text{OD of positive control }-\text{OD of negative control}}. \end{array}$$
(2) For the antibody titer, log_10_ (titer) = 0.97 × log_10_ (S/P) + 3.449; titer = 10^log^_10_^(titre)^.

Results were interpreted as follows (see Table 1):

### 2.7. Statistical Analysis

Data from the field and laboratory were combined in a Microsoft Excel 2013 (Los Angeles, CA, USA) spreadsheet, which was subsequently thoroughly verified for mistakes and inconsistencies and sorted, coded, and tested for integrity. Finally, the data were exported to STATA-IC-13 (Stata Corp, 4905 Lakeway Drive, College Station, Texas 77845, USA) for statistical analysis.

#### 2.7.1. Descriptive Analysis

Descriptive analysis was performed to enumerate the frequencies and percentages of the results to determine the demographic characteristics. The seroprevalence of IBDV antibodies in backyard chickens was determined at the birth level and epidemiological factors. Furthermore, a 95% confidence interval (CI) for IBDV seropositivity was calculated to determine the estimated values.

#### 2.7.2. Risk Factor Analysis

Risk factor analysis (both uni- and multivariable) was conducted to determine the association between the seroprevalence of IBDV antibodies resulting from the ELISA test and different important epidemiological risk factors.

*(1) Univariable analysis*. Eight important epidemiological factors were considered for univariable analysis: (1) location (five Upazilas of the Mymensingh district); (2) variation in the ages of backyard chickens; (3) whether chickens were male or female; (4) whether chickens were healthy or unhealthy; (5) whether chickens were nondescriptive indigenous or crossed; (6) whether chickens were reared in the house or imported and/or brought from markets; (7) whether chickens were reared by a free-ranging system or in a separate house; and (8) whether the conditions of the farming houses were good, moderate, or poor.

A univariate chi-square (two-tailed) test was used to determine the potential link between the seroprevalence of IBDV and distinct epidemiological risk factors. A statistically significant *p* value was less or equal to 0.05 (*p* ≤ 0.05).

*(2) Multivariable analysis*. First, epidemiological risk factors with a significant association (*p* ≤ 0.05, univariable two-tailed chi-square test) were deemed suitable for multivariable analysis. Subsequently, backward stepwise logistic regression was performed to fit the model. Initially, a full model was conducted, with variables kept only if the likelihood ratio test resulted in *p* ≤ 0.05 Interactions between biologically justifiable epidemiological risk factors were also examined, and those that were significant (*p* ≤ 0.05) were preserved in the final stage.

A two-tailed *p* value was used to examine the collinearity between categorical components using “Fishers” exact test. Two factors were considered collinear if their *p* values were <0.05. The sensitivity of the resulting model was then checked for goodness-of-fit using the Hosmer–Lemeshow test, and the receiver operating characteristic curve was used to obtain the postestimate of predictive ability [31]. The results are presented as odds ratio (OR), *p* value, and 95% CI for each adjusted predictor variable.

## 3. Results

### 3.1. Sample Description

A total of 460 backyard chicken blood samples were collected, among which, location-wise, Mymensingh Sadar Upazila (41.3%) accounted for the most collected chicken blood samples; the highest blood samples were obtained from females (76.1%); 4–6 week (55.7%) age groups; healthy (87.0%), nondescriptive (86.9%) indigenous chickens; and chickens reared in the home (84.8%), free-ranging housing system (65.2%), and moderate hygienic-conditioned houses (45.7%) (Table 2).

### 3.2. Seroprevalence

Overall, 83.4% (384/460, 95% CI: 79.8%–86.6%) of the backyard chicken blood samples were found to be positive for IBDV antibodies; among them, the highest seroprevalence of IBDV antibodies in backyard chickens was recorded in Fulbaria Upazila (88.8%, 80/90, 95% CI: 80.5%–94.0%) among other Upazilas of the Mymensingh district; in males (98.6%, 345/350.95% CI: 96.6%–99.4%); unhealthy (95.0%, 57/60, 95% CI: 85.7%–98.8%) backyard chickens; chickens that were imported and/or brought from the marketplaces (85.7%, 60/70, 95% CI: 75.4%–92.2%); and chickens reared in free-ranging housing systems (93.3%, 280/300, 95% CI: 89.8%–95.6%) and poor hygienic-conditioned houses (98%, 147/150, 95% CI: 94.0%–99.5%). The overall seroprevalence of the IBDV antibodies is shown in Table 3.

### 3.3. Risk Factor Analysis

#### 3.3.1. Univariate Association Analysis for the Risk Factors

Univariable analysis revealed a strong significant association between the seroprevalence of IBDV antibodies and different important epidemiological risk factors, such as sex (*p* < 0.001), age groups (*p* < 0.001), health status (*p*=0.01), breed type (*p* < 0.001) of backyard chickens, chicken housing system (*p* < 0.001), and hygienic conditions of chicken houses (*p* < 0.001). The results of univariate analysis are presented in Table 3.

#### 3.3.2. Multivariable Regression

Risk factors that were statistically significant (*p* < 0.05) in the univariable analysis (i.e., sex, age, health status, breed type, housing system, and hygienic conditions) were subjected to multivariable regression analysis. The results of the multivariable analysis between the seroprevalence of IBDV antibodies and the significant risk factors are presented in Table 4. Using multivariable regression analysis, female backyard chickens had 125.6 times the odds (OR = 125.6, 95% CI: 47.8%–329.8%, *p* < 0.001) of seroprevalence of IBDV antibodies than those of male backyard chickens; 4–6 weeks (*O* = 40.6, 95% CI: 18.4–89.5, *p* < 0.001) and 7–12 weeks (OR = 10, 95% CI: 5.0–19.9, *p* < 0.001) age groups of backyard chickens had 40.6 and 10 times the odds of seroprevalence of IBDV antibodies compared with the 1–3 week age group of backyard chickens. Similarly, the significant effect of health status on the seroprevalence of IBDV antibodies was 4.2 times in unhealthy backyard chickens (OR = 4.2, 95% CI: 1.2–13.9, *p*=0.01) than that in healthy backyard chickens. In addition, crossbred backyard chickens (OR = 6.5, 95% CI = 1.5–27.5, *p*=0.01) had a significant association (6.5 times) with the occurrence of IBDV antibodies against IBD in nondescriptive indigenous backyard chickens. Similarly, chickens reared in separate houses had 0.1 times the odds (OR = 0.1, 95% CI: 0.07–0.23, *p* < 0.001) of seropositivity for IBDV antibodies compared with chickens reared in free-ranging housing systems, chickens reared in poor hygienic-conditioned houses had 31.3 times (OR = 31.3, 95% CI: 9.3–105.2, *p* < 0.001), and chickens reared in moderate-conditioned houses had 3.3 times (OR = 3.3, 95% CI: 1.9–5.7, *p* < 0.001) higher odds than backyard chickens reared in good-conditioned houses.

## 4. Discussion

The introduction and outbreak of viral diseases in backyard chicken populations have hampered the growth of rural economies. IBD is recognized as one of the most serious diseases in chickens in terms of morbidity and economic losses [13]. Therefore, isolating and identifying IBDV in backyard chickens is critical. Few studies on broiler and layer birds have been conducted in Bangladesh [32, 33, 34]; however, there is a lack of seroepidemiological detection of IBDV in backyard chickens. Using indirect ELISA, the current cross-sectional serological study was conducted to detect IBDV antibodies in backyard chickens in the Mymensingh district of Bangladesh.

In this study, 83.4% of the selected samples were confirmed positive for IBDV infection in backyard chickens. Here, we obtained confirmation from poultry owners at the time of sample collection that chickens had never been vaccinated against any poultry diseases. Consequently, the occurrence of IBD antibodies in the present study provides evidence that backyard chickens were exposed to natural IBDV infection. Our current findings were similar to those of previous studies conducted globally, for example, 86% by Bolfa et al. [34] in St. Kitts, 83.1% by Jenbreie et al. [35], and 83% by Tadesse and Jenbere [20] in Ethiopia. Converse findings were also recorded by different earlier studies; for example, a higher prevalence was detected by Chaka et al. [36] in Ethiopia with 91.9% seropositivity; lower prevalence was determined by Jordan et al. [37] in Trinidad and Tobago, Abdeta et al. [38] in Western Ethiopia, and Lemma et al. [39] in Eastern Ethiopia with 67.5%, 66.93%, and 51.7% seropositive rates of IBDV antibodies, respectively. Disparities in several risk factors, such as geographical distribution, sample size, sample collection procedure, sample processing system, and sensitivity and specificity of the detection tools, could lead to significant variations between the current study and earlier investigations. Furthermore, an unhygienic management system, lack of nutritional supplements, and direct interaction with other exotic chickens may play a role in the higher prevalence of IBDV antibodies in backyard chickens [24]. The high seroprevalence of IBD in research areas with no apparent mortality may be due to less pathogenic IBDV. Moreover, backyard chickens may have been infected with IBDV as adults, and at that stage, they were seroconverted, with no obvious clinical diseases. Owing to the high seropositivity and low mortality of infected chickens, they may be genetically resistant to Bangladeshi backyard chickens. However, these possible reasons are difficult to demonstrate using only serological observations, and further studies, such as molecular and genome sequencing techniques, should be conducted to investigate them properly.

Location-wise, Fulbaria Upazila had the highest seropositivity (88.8%) of IBDV antibodies, whereas Trishal Upazila had the lowest (70.0%); however, no significant variations among the selected Upazilas of the Mymensingh district were noted. Female chickens had a significantly higher seroprevalence rate of IBDV antibodies than males, which is consistent with previous global studies [10, 35, 39, 40]. Physiological and immunological variations between the sexes may support our findings [41]. In addition, compared with males, females have a more susceptible reproductive system and habits, which may increase the risk of infection [41]. The presence of IBD in both female and male chickens in the current study highlights the critical importance of routine vaccination to prevent the spread of IBDV.

Regarding age groups, backyard chickens within 4–6 weeks of age were exposed to a significantly higher seroprevalence of IBDV than other age groups, which is supported by previous studies [20, 35]. This could be due to variations in the development of the bursa of Fabricius, which usually matures and reaches its maximum size at the age of 3–6 weeks [42]. In addition, the bursa is believed to be the site of IBDV proliferation, and chickens aged 3–6 weeks are more susceptible to IBD [42]. Furthermore, people usually rear backyard chickens of different ages in a flock using a chicken production system that ultimately affects chickens of different ages.

IBDV infection was significantly more common in crossbred backyard chickens than in nondescriptive indigenous backyard chickens. Currently, crossbreeding is being rigorously used to improve the productivity and contribution of chickens in Bangladesh. However, crossed chickens subjected to inbreeding or unethical breeding to make more profit quickly may be more vulnerable to IBD, which may be an attributable cause of increased IBDV seroprevalence.

Unhealthy backyard chickens had a significantly higher (*p* < 0.05) occurrence of IBDV infection; however, the source of the backyard chickens had no significant association (*p* > 0.05). The current findings could be attributed to IBDV being transmitted directly or indirectly from other birds to backyard chickens via open-market purchases. In addition, unhealthy chickens were more likely to be seropositive for IBDV infection, possibly owing to a lack of proper immunity.

The rearing system has a significant impact on the occurrence of infectious diseases, including IBD, in backyard chickens. Housing system had a substantial impact (*p* < 0.05) on the prevalence of IBD in backyard chickens, with free-range chickens showing significantly higher IBDV antibody seropositivity. This could happen because of openly moving backyard chickens across the village, putting them in direct contact with vulnerable wild and exotic birds as well as freely wandering rodents. Hygienic conditions in farming houses can also contribute to the incidence of IBD in backyard chickens.

In this study, the seroprevalence of IBDV antibodies was significantly higher (*p* < 0.05) in backyard chickens reared in poor-conditioned houses than in those reared in moderate- and good-conditioned houses. In Bangladesh, most backyard chicken farmers feed birds on the ground, and only a few farmers use feeders and drinkers to provide feed and water to birds [43]. Moreover, influencing factors, including overcrowding of chickens, irregular cleaning, poor ventilation systems, and poor litter conditions, may be the cause of increased seropositivity of IBDV antibodies in poor-conditioned houses.

## 5. Conclusions

The presence of IBDV antibodies in the sera of backyard chickens in the present study suggests natural exposure and horizontal transmission of IBDV from backyard chickens to other chickens, which poses a serious threat to the poultry sector. Furthermore, the significant association between the occurrence of IBDV infection and age, sex, health status, breed types, housing system of backyard chickens, and hygienic conditions indicated the significance of strict biosecurity measures for disease prevention and control. Further research on the detection of IBDV prevalence and virulence at the molecular and genome sequencing level could provide an accurate picture of the disease's current state in backyard chickens in Bangladesh.

## Figures and Tables

**Table 1:** Interpretation of IBD immune status based on S/P value and ELISA antibody titre.

S/*P* value	ELISA antibody titre	IBD immune status
S/P ≤ 0.3	Titre ≤875	Negative
S/P > 0.3	Titre >875	Positive

S/*P* = sample-to-positive, ELISA = enzyme-linked immunosorbent assay, IBD = infectious bursal disease.

**Table 2:** Frequency distribution of demographic characteristics (*N* = 460).

Variable	Category	Frequency (n)	Percentage (%)
Location	Mymensingh Sadar	190	41.3
	Fulbaria	90	19.6
	Muktagacha	60	13.0
	Phulpur	70	15.2
	Trishal	50	10.9

Sex	Male	110	23.9
	Female	350	76.1

Age	1–3 weeks	60	13.0
	4–6 weeks	256	55.7
	7–12 weeks	144	31.3

Status of health	Healthy	400	86.9
	Unhealthy	60	13.1

Breed type	Indigenous	400	87.0
	Crossbreed	60	13.0

Source	Home-reared	390	84.8
	Brought-in	70	15.2

Housing system	Free-ranging	300	65.2
	Separate house	160	34.8

Hygienic condition	Good	100	21.7
	Moderate	210	45.7
	Poor	150	32.6

**Table 3:** Univariate association between seroprevalence of IBDV using ELISA with different factors.

Variable	Category	Positive, *n* (%)	95% CI (%)	*p* value
Location	Mymensingh Sadar	160 (84.2)	78.3–88.7	0.06
	Fulbaria	80 (88.8)	80.5–94.0	
	Muktagacha	49 (81.6)	69.9–89.6	
	Phulpur	60 (85.7)	75.4–92.2	
	Trishal	35 (70.0)	56.1–80.9	

Sex	Male	39 (35.4)	27.1–44.7	0.00
	Female	345 (98.6)	96.6–99.4	

Age	1–3 weeks	20 (33.3)	22.6–45.9	0.00
	4–6 weeks	244 (95.3)	91.9–97.3	
	7–12 weeks	120 (83.3)	76.3–88.6	

Status of health	Healthy	327 (81.7)	77.6–85.2	0.01
	Unhealthy	57 (95.0)	85.7–98.8	

Breed type	Indigenous	326 (81.5)	77.3–85.0	0.00
	Crossbreed	58 (96.6)	87.9–99.7	

Source	Home-reared	324 (83.1)	79.0–86.4	0.58
	Brought-in	60 (85.7)	75.4–92.2	

Housing system	Free-ranging	280 (93.3)	89.8–95.6	0.00
	Separate house	104 (65.0)	57.3–71.9	

Hygienic condition	Good	61 (61.0)	51.1–69.9	0.00
	Moderate	176 (83.8)	78.1–88.2	
	Poor	147 (98.0)	94.0–99.5	

Here, a *p* value ≤ 0.05 was considered statistically significant, CI = confidence interval.

**Table 4:** Multivariable logistic regression model between binary response variable results and the selected factors (*N* = 460).

Variable	Category	OR	95% CI	*p*-value
Sex	Male	Ref		
	Female	125.6	47.8–329.8	0.00

Age	1–3 weeks	Ref		
	4–6 weeks	40.6	18.4–89.5	0.00
	7–12 weeks	10	5.0–19.9	0.00

Status of health	Healthy	Ref		
	Unhealthy	4.2	1.2–13.9	0.01

Breed type	Indigenous	Ref		
	Crossbreed	6.5	1.5–27.5	0.01

Housing system	Free-ranging	Ref		
	Separate house	0.1	0.07–0.23	0.00

Hygienic condition	Good	Ref		
	Moderate	3.3	1.9–5.7	0.00
	Poor	31.3	9.3–105.2	0.00

Here, a *p* value ≤0.05 was considered statistically significant, OR = odds ratio, CI = confidence interval.

## Data Availability

The datasets used and/or analyzed during the current study are available from the corresponding author upon request.
